# Thermal and mechanical quantitative sensory testing in chinese patients with burning mouth syndrome – a probable neuropathic pain condition?

**DOI:** 10.1186/s10194-015-0565-x

**Published:** 2015-09-24

**Authors:** Xueyin Mo, Jinglu Zhang, Yuan Fan, Peter Svensson, Kelun Wang

**Affiliations:** Jiangsu Key Laboratory of Oral Diseases, Nanjing Medical University, 136 Hanzhong Road, Nanjing, 210029 China; Hangzhou ivy dental clinic Co., Limited, 156 Tianmushan road, Hangzhou, 310000 China; Orofacial Pain and TMD Research Unit, Institute of Stomatology, Affiliated Hospital of Stomatology, Nanjing Medical University, 136 Hanzhong Road, Nanjing, 210029 China; Section of Orofacial Pain and Jaw Function, School of Dentistry, Aarhus University, Aarhus, Denmark; Department of Dental Medicine, Karolinska Institutet, Huddinge, Sweden; Center for Sensory-Motor Interaction (SMI) Aalborg University and Stomatological School and Hospital,Nanjing Medical University, Nanjing, PR China

**Keywords:** Burning mouth syndrome, Quantitative sensory testing, Pathophysiology

## Abstract

**Background:**

To explore the hypothesis that burning mouth syndrome (BMS) probably is a neuropathic pain condition, thermal and mechanical sensory and pain thresholds were tested and compared with age- and gender-matched control participants using a standardized battery of psychophysical techniques.

**Methods:**

Twenty-five BMS patients (men: 8, women: 17, age: 49.5 ± 11.4 years) and 19 age- and gender-matched healthy control participants were included. The cold detection threshold (CDT), warm detection threshold (WDT), cold pain threshold (CPT), heat pain threshold (HPT), mechanical detection threshold (MDT) and mechanical pain threshold (MPT), in accordance with the German Network of Neuropathic Pain guidelines, were measured at the following four sites: the dorsum of the left hand (hand), the skin at the mental foramen (chin), on the tip of the tongue (tongue), and the mucosa of the lower lip (lip). Statistical analysis was performed using ANOVA with repeated measures to compare the means within and between groups. Furthermore, Z-score profiles were generated, and exploratory correlation analyses between QST and clinical variables were performed. Two-tailed tests with a significance level of 5 % were used throughout.

**Results:**

CDTs (*P* < 0.02) were significantly lower (less sensitivity) and HPTs (*P* < 0.001) were significantly higher (less sensitivity) at the tongue and lip in BMS patients compared to control participants. WDT (*P* = 0.007) was also significantly higher at the tongue in BMS patients compared to control subjects . There were no significant differences in MDT and MPT between the BMS patients and healthy subjects at any of the four test sites. Z-scores showed that significant loss of function can be identified for CDT (Z-scores = −0.9±1.1) and HPT (Z-scores = 1.5±0.4). There were no significant correlations between QST and clinical variables (pain intensity, duration, depressions scores).

**Conclusion:**

BMS patients had a significant loss of thermal function but not mechanical function, supporting the hypothesis that BMS may be a probable neuropathic pain condition. Further studies including e.g. electrophysiological or imaging techniques are needed to clarify the underlying mechanisms of BMS.

## Background

Burning mouth syndrome (BMS) is characterized by the presence of a persistent burning sensation of the oral mucosa in the absence of clinical or laboratory data to explain these symptoms [1, 2]. Thus, it is a chronic orofacial pain condition, unaccompanied by obvious mucosal lesions or other evident clinical signs upon examination [1–3]. BMS occurs more commonly in menopausal women and often affects the tongue (particularly the tip and lateral borders), lips, and hard and soft palates [4].

The pathophysiology of BMS remains an enigma. Various factors are related to its mechanisms, and overall this syndrome has been divided into local, systemic and psychological types [5, 6]. Several studies have been performed to examine the possible underlying mechanisms of BMS [7–9]. Indeed, neurophysiological studies have elucidated that several neuropathic mechanisms, mostly subclinical, act at different levels of the neuraxis and may contribute to the pathophysiology of primary BMS [3, 6]. All these research methods have now been applied in the study of BMS patients, which, together with more rigorous clinical diagnostic definitions, has resulted in progress in the understanding of the pathophysiological mechanisms underlying BMS [3].

As a reliable, non-invasive psychophysical tool for evaluating the conscious perception of different standardized stimuli, quantitative sensory testing (QST) has been used to investigate small and large nerve fiber function [10, 11]. However, several studies using QST in BMS patients have produced conflicting results. Jääskeläinen et al. [12] reported differences in thermal pain thresholds on the tongue in patients with BMS when compared with healthy control subjects. In contrast, Kaplan et al. [13] and Grushka et al. [7] found that BMS was not associated with abnormal thermal and pain thresholds. These contradictory findings might be attributable to methodological differences with regard to psychophysical techniques, devices and clinical characteristics of the BMS populations.

The primary aim of our study was to compare thermal and mechanical sensory and pain thresholds in BMS patients and their age- and gender-matched controls to investigate a probable neuropathic basis of BMS. The secondary aim was to perform explorative correlation analyses between QST variables and clinical characteristics.

## Methods

### Participants

Twenty-five BMS patients (men: 8, women: 17, age: 49.5 ± 11.4 years) and 19 age- and gender-matched healthy control participants were included in the study. None of the participants had prior experience with QST methods.

The inclusion criteria of BMS patients were according to the ICHD-3 classification as follows: 1. superficial intraoral pain for more than 3 months; 2. a persistent (more than 2 h/day) and burning quality of the pain; age from 30 to 68 years; 3. no visible clinical changes of the oral mucosa (redness, swelling, lichen planus and ulcer). The inclusion criterion of the control participants was self-reported health without a history of any type of orofacial pain problems. The exclusion criteria for all participants were as follows: systemic factors known to be related to BMS/orofacial pain, a history of systemic diseases or mental disorders, the presence of any acute or chronic pain conditions in the head, neck, face and upper limb region, ongoing dental treatment, taking pain medication, antidepressants or non-steroidal anti-inflammatory drugs (NSAIDs) in the last month, and the current use of caffeine within 24 h of the day of testing. Specifically, BMS patients were ruled out for candida infections by candida culture, vitamin B12 deficiency, folic acid deficiency, diabetes by blood tests and hyposalivation by the spit method for 5 min.

The protocol was approved by the local ethical committee of Nanjing Medical University. P.C. with approval number (No:PJ2013-013-04). Informed consent in accordance with the Helsinki II declaration was obtained from all participants prior to inclusion.

### Study design

Thermal and mechanical sensory testing was performed at the following four sites for all participants: tip of the tongue (tongue), mucosa of the lower lip (lip), skin of the mental foramen (V3), and dorsum of the left hand (hand, C7, spinal region). All participants were tested at the four sites in a randomized manner by one examiner (Xueyin Mo) who had been trained extensively in the use of QST by Wang K and Svensson P.

### Thermal detection thresholds and thermal pain thresholds

The thermal tests were performed using a computerized thermal stimulator (MEDOC TSA-2001 apparatus, Medoc Ltd, Ramat-Yishai, Israel). Two different thermodes were used for the assessments. The contact area of the extra-oral thermode was 30 × 30 mm and that of the intra-oral thermode was 6 × 6 mm.

Cold and warm detection thresholds (CDT, WDT) were measured first, followed by cold and heat pain thresholds (CPT, HPT). The mean thresholds of three consecutive measurements were calculated. The temperature of the thermode started from a baseline of 32 **°**C for the extra-oral site and 37 **°**C for the intra-oral sites and heated-up or cooled-down at a rate of 1 **°**C/s to the lower limits of 0 **°**C or upper limits of 50 **°**C. The participants were instructed to press a button on the computer mouse as soon as they perceived the respective thermal sensation of cold, warm, cold pain, or heat pain following the instructions developed by the German Research Network on Neuropathic Pain (DFNS). The procedure then ended, and the temperature returned to baseline at a rate of 1 **°**C/s. The participants were instructed not to look at the computer screen at any time during the testing procedures.

### Mechanical detection threshold and mechanical pain threshold for pinprick stimuli

Mechanical detection thresholds (MDT) were measured using standardized Semmes-Weinstein monofilaments with 20 different diameters (North Coast Medical, Canada). The number of each filament (1.65 to 6.65) corresponds to a logarithmic function of the equivalent forces of 0.008 to 300 g. The filament was applied vertically on the test sites, and pressure was applied slowly until the filament bowed with a total contact time of approximately 1 s. To prevent filament slippage, intra-oral examination sites were dried with gauze before testing [14, 15].

To detect the mechanical pain threshold (MPT), weighted pinprick stimuli delivered with a custom-made set of seven pinprick stimulators (Aalborg University, Denmark) were used [16]. Each stimulator had a flat contact surface of 0.2 mm that exerted forces of 8–512 mN. All pinprick tests were made with the stimulator perpendicular to the examination site and in a vertical position with a contact time of 1 s. MDT and MPT were measured using the “method of limits” technique described by Baumgartner [17]. Five threshold measurements were made, applying a series of ascending and descending stimulus intensities. One threshold value was determined by calculating the geometric mean of these five series.

### Clinical characteristics

The present pain intensity was rated on a numerical rating scale (NRS) from 0 = no pain to 10 = worst pain imaginable; the duration of BMS pain (months) and the location of BMS (questionnaires) were also determined. Furthermore, all participants were screened for signs of major depression with the Zung Self-Rating Depression Scale, which includes 20 items [18]. The Zung Self-Rating Depression Scale is a short, self-administered survey that quantifies the depressed status of a patient. The Chinese version has been validated and used in previous studies [19]. Scores on the test range from 20 through 80. The scores fall into the following four ranges: 20–44 normal; 45–59 mildly depressed; 60–69 moderately depressed; and 70 and above severely depressed.

### Statistical analysis

Descriptive statistics were used to summarize all measurements. Thermal and pain thresholds were expressed as the means ± SD. The necessary logarithmic transformation was performed to secure normal distribution of the data set. The data was first analyzed using a 2-way ANOVA with repeated analysis of variance, with the group as between-subject factors and the test site as within-subject factor. A Bonferroni test was employed for post-hoc comparisons.

Second, to demonstrate the degree of the differences between the BMS patients and controls groups independently of the different units of the QST parameters, a Z-score transformation was performed for all QST variables to provide a somatosensory profile [20]. A Z-score, which is a score indicating how many standard deviations an observation isfrom the mean of the distribution, could then be calculated. The data from the healthy control group were set as the reference data for the Z-score transformation, and all the patient data were transformed based on the reference data. The formula is as follows: Z-score = (value _patients_–value _controls_)/SD _controls_.

Finally, explorative correlations analyses were performed between the QST variables and clinical characteristics, such as pain intensity, pain duration and depression scores, using Spearman tests. All statistical calculations were performed using the Statistical Package for Social Sciences, version 20 (SPSS, IBM). The significance level was set at 0.05.

## Results

All participants were able to understand the instructions and cooperate during the QST. The healthy control participants were age- and gender-matched with the BMS patients (see Table 1). The clinical characteristics of the BMS patients are shown in Table 1. Notably, the depression scores of all participants were less than 44, i.e., within the normal range. The absolute values of all variables, CDT, WDT, CPT, HPT, MDT and MPT, in the two groups at the four test sites are shown in Table 2.

**Table 1:** Clinical characteristics of BMS patients and healthy control participants

	BMS	Controls
	(*n* = 25)	(*n* = 19)
Age (years)	49.5 ± 11.4	47.7 ± 12.4
Sex (% females)	68 %	63 %
Pain duration (month)	12.7 ± 8.1	/
Pain intensity (0–10 NRS)	5.4 ± 1.6	/
Location (%)	Tongue 80 %; lip 28 %; palate 12 %	/
Depressive symptoms (0-80*)	37.1 ± 2.1	34.3 ± 2.3

*Zung Self-Rating Depression Scale

NRS = numerical rating scale

**Table 2:** Mean values and standard deviation for thermal and mechanical QST parameters at four sites in BMS patients and healthy subjects

	BMS patients				Healthy subjects			
	Tongue	Lip	Chin	Hand	Tongue	Lip	Chin	Hand
CDT (°C)	34.0 ± 1.3	33.8 ± 1.6	31.0 ± 0.4	30.2 ± 0.7	35.0 ± 1.1	35.1 ± 0.7	31.0 ± 0.5	29.9 ± 1.1
WDT (°C)	39.4 ± 1.0	39.4 ± 2.1	33.4 ± 0.6	34.4 ± 0.8	38.3 ± 1.3	38.7 ± 1.5	33.5 ± 1.1	34.6 ± 1.0
CPT(°C)	14.9 ± 5.6	13.3 ± 5.4	15.8 ± 4.6	14.3 ± 4.2	15.7 ± 4.1	14.4 ± 4.4	16.0 ± 5.1	15.5 ± 5.2
HPT (°C)	45.3 ± 2.7	45.8 ± 2.2	43.8 ± 1.8	43.5 ± 1.8	42.4 ± 2.0	43.2 ± 1.9	43.0 ± 2.1	42.9 ± 2.0
MDT	1.7 ± 0.2	1.7 ± 0	1.7 ± 0	1.9 ± 0.3	1.8 ± 0.2	1.7 ± 0	1.7 ± 0	2.0 ± 0.3
MPT (mN)	86.7 ± 12.8	97.3 ± 25.3	170.3 ± 30.1	331.2 ± 94.0	95.3 ± 20.2	100.1. ± 27.8	168.1 ± 32.5	322.3 ± 93.2

*CDT* cold detection threshold, *WDT* warm detection threshold, *CPT* cold pain threshold, *HPT* heat pain threshold, *MDT* mechanical detection threshold, *MPT* mechanical pain threshold

### CDT and CPT

At the tongue and lip, the CDTs in the BMS patients were significantly lower (less sensitivity) than in the healthy subjects (*P* = 0.02), there were no significant differences between the two groups at the hand and V3 sites (Table 3).

**Table 3:** Test of within-subject and between-subject effects for CDT, WDT, CPT, HPT ,MDT and MPT between BMS patients and healthy subjects at four sites by 2-way ANOVAs with repeated measures

	Tongue	Lip	Chin	Hand
CDT	0.020*	0.002*	0.900	0.080
WDT	0.007*	0.200	0.800	0.500
CPT	0.400	0.060	0.800	0.900
HPT	<0.001*	<0.001*	0.200	0.300
MDT	0.800	0.200	0.200	0.200
MPT	0.200	0.300	0.060	0.800

*indicate significant difference

### WDT and HPT

At the tongue and lip, the HPTs in the BMS patients were higher (less sensitivity) than in the healthy subjects (*P* <0.001). Furthermore, at the tongue, the WDTs in the BMS patients were also significantly higher (less sensitive) compared to control subjects (*P* = 0.007), as shown in Table 3. There were no significant differences between the two groups at the hand and V3 sites (Table 3).

### MDT and MPT

For the mechanical stimuli, there were no significant differences between the BMS patients and healthy subjects at any of the four test sites (Table 3).

### Z-score profiles

**Fig. 1:**
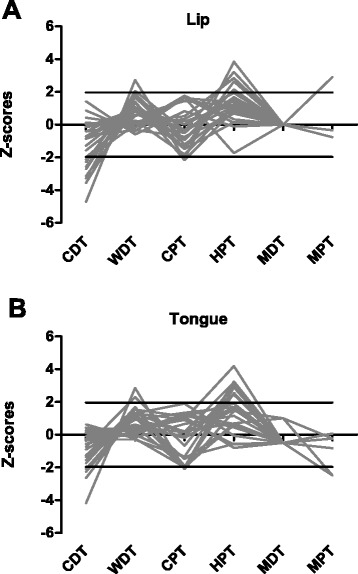
The individual z-score profiles of the 25 patients with Burning mouth syndrome (BMS). **a** Z-scores of lip in BMS patients; **b** Z-scores of tongue in BMS patients; CDT = cold detection threshold; WDT = warmth detection threshold; CPT = cold pain threshold; HPT = heat pain threshold; MDT = mechanical detection threshold; MPT = mechanical pain threshold

Figure 1 shows the Z-scores for all the QST variables. Z-scores were calculated for tongue and lip. Significant loss of function (Z-scores < −1.96) can be identified for CDT and HPT (Z-scores > 1.96). Abnormal Z-scores were detected in nine patients for CDT (36 %) and in 7 patients for HPT (28 %) at the lip. Likewise, abnormal Z-scores were detected in 4 patients for CDT (16 %) and in eight patients for HPT (32 %) at the tongue.

### Correlation analyses

The explorative correlation analyses did not demonstrate any significant associations amongst any of the QST variables or the clinical characteristics (all R < 0.40; *P* > 0.05) (data not shown).

## Discussion

In this study performed in Chinese patients fulfilling the ICHD-3 criteria for BMS, thermal and mechanical QSTs were measured based on previous experience, i.e., we focused on 4 thermal and 2 mechanical psychophysical tests instead of the complete DFNS protocol which includes a total of 13 different tests. We found that BMS patients had lower cold detection and pain thresholds (less sensitivity) and higher warm detection and pain thresholds (less sensitivity) at the tongue and lip than healthy participants. In addition, there were no significant differences in mechanical detection and pain thresholds between the BMS patients and healthy participants at any test site. These findings with a localized loss of thermal function in the BMS patients further support the hypothesis that BMS could be a neuropathic pain condition with involvement of peripheral and/or central pain mechanisms.

An increasing number of investigations suggest pathophysiological alterations at different levels of the neuroaxis, either alone or simultaneously within the peripheral or central nervous system, in the etiopathogenesis of primary BMS. However, some studies using QST to assess this phenomenon have produced conflicting results. Grushka et al. [7] performed the first systematic psychophysical study on BMS patients using QST methods to investigate tactile and thermal sensory modalities in the orofacial region, including the tongue mucosa. They did not find a difference between BMS and control subjects in the detection thresholds of any of the tested sensory modalities. Kaplan et al. [13] found that BMS was not associated with abnormal thermal and pain thresholds. In contrast, Jääskeläinen et al. and Puhakka et al. showed significant changes in HPT at the tip of the tongue of BMS patients [21, 22]. A QST study utilizing an argon laser stimulator demonstrated increased WDT and HPT (hypoesthesia and hypoalgesia, i.e., negative signs) on the tongue of BMS patients compared to healthy control participants [8]. Some differences exist between Grushka and Kaplan’s studies and our own study, such as higher baseline probe temperature and the thermode size. The thermodes in their study were larger (2 × 2 cm) and may be less suitable for the study of the small trigeminal distributions [14, 21, 22]. Furthermore, there may be differences in the BMS populations; however, the present study adhered to the proposed clinical criteria by the ICHD-3.

As a measure of Aδ-fiber function, CDT, CPT, HPT and MPT were assessed. C-fiber function was tested by assessing the thresholds for WDT. The present study found that BMS patients had hypoesthesia at the tongue and lip. However, the present study did not find any localized loss of mechanical function in BMS patients, indicating that the small-fiber neuropathy may only, or predominately, involve the small C and A-δ nerve fibers in BMS patients. Neurophysiological testing and biopsies of the tongue have indicated that there are peripheral nerve changes with abnormal perception of temperature but central nervous system changes have also been noted using fMRI testing [23]. Lauria et al. [24] found that BMS patients have a trigeminal small-fiber sensory neuropathy affecting the tongue, characterized by a significant loss of epithelial and sub-papillary nerve fibers, as well as diffuse axonal derangement, through tongue mucosal biopsies and immune-histochemical studies. Previous studies have shown that epidermal nerve fibers have synaptic contacts with the taste buds of the fungiform papillae [25] and that their stimulation can induce a burning sensation and affect gustatory perception [26], which might explain why dysgeusia is a frequent symptom in BMS patients. Furthermore, Yilmaz et al. and Beneng et al., using specific immunohistochemical staining of tongue mucosal biopsies, have revealed significant increases in the expression of NGF, TRPV1 ion channels, and P2X3 receptors within the surviving subepithelial nerve fibers of BMS patients [27, 28]. These factors have been associated with hypersensitivity and neuropathic pain symptoms in various experimental models and human pain conditions, and they could perhaps be related to the burning pain symptoms in BMS patient as well [27, 28].

Although several neurophysiological studies have suggested that the central and/or peripheral nervous systems are implicated in the pain of BMS [8, 21, 22, 24, 27, 28], the pathophysiology of BMS is still complex. In a large number of patients, it probably involves multiple interactions between local, systemic and psychological factors. Frequently, several factors coincide, increasing the harmful effect on the mucosa, whether perceptible or not by the observer.

It should be noted that the ICHD-3 criteria specifically mention a lack of clinical sensory abnormalities in BMS patients. However, the present study clearly demonstrates a loss of thermal function in a group of BMS patients who otherwise adhered strictly to the ICHD-3 criteria. Furthermore, several other studies [8, 22, 24], although not all studies [7, 13], have demonstrated differences in somatosensory function between BMS patients and control subjects; thus, the criteria in the ICHD-3 may need to be reconsidered.

The main limitation of this study is that the sample size is relatively small, and due to time constraints, we only chose the one most likely to demonstrate a difference in QST parameters rather than the entire DFNS QST battery. The strengths of the present study include the fact that compared to other studies; we examined four test sites, adding the lower lip site which often is associated with burning pain in BMS patients. Furthermore, we included an extraoral control site, in strict accordance with the standardized QST guidelines [29] and the examiner (Xueyin Mo) who had been trained extensively in the use of QST by Wang K and Svensson P. In addition, the findings are robust because in addition to ANOVA, Z-score profiles were generated and exploratory correlation analyses between QST and clinical variables were performed.

## Conclusion

The present study in Chinese patients with BMS pain convincingly demonstrated a loss of thermal function but not mechanical function when compared to age- and gender matched control participants. No correlations were found between QST variables and clinical characteristics. It can be proposed that BMS appears to fulfill the criteria for a probable neuropathic pain condition. Further studies combining QST, electrophysiology, biopsies and imaging tests will be needed to substantiate this hypothesis.

### Ethical committee

All experiments were performed in accordance with the guidelines of the Nanjing Medical University ethics committee (No: PJ2013-013-04).

## References

[1] Torgerson RR (2010). Burning mouth syndrome. Dermatol Ther.

[2] Patton LL, Siegel MA, Benoliel R, De Laat A (2007). Management of burning mouth syndrome: systematic review and management recommendations. Oral Surg Oral Med Oral Pathol Oral Radiol Endod.

[3] Jääskeläinen SK (2012). Pathophysiology of primary burning mouth syndrome. Clin Neurophysiol.

[4] Grushka M, Ching V, Epstein J (2006). Burning mouth syndrome. Adv Otorhinolaryngol.

[5] López-Jornet P, Camacho-Alonso F, Andujar-Mateos P, Sanchez-Siles M, Gomez-Garcia F (2010). Burning mouth syndrome:an update. Med Oral Patol Oral Cir Bucal.

[6] Forssell H, Jääskeläinen S, List T, Svensson P, Baad-Hansen L (2015). An update on pathophysiological mechanisms related to idiopathic oro-facial pain conditions with implications for management. J Oral Rehabil.

[7] Grushka M (1987). Clinical features of burning mouth. Oral Surg Oral Med Oral Pathol.

[8] Svensson P, Bjerring P, Arendt-Nielsen L, Kaaber S (1993). Sensory and pain thresholds to orofacial argon laser stimulation in patients with chronic burning mouth syndrome. Clin J Pain.

[9] Heckmann SM, Heckmann JG, Hilz MJ, Popp M, Marthol H (2001). Oral mucosal blood flow in patients with burning mouth syndrome. Pain.

[10] Shukla G, Bhatia M, Behari M (2005). Quantitative thermal sensory testing value of testing for both cold and warm sensation detection in evaluation of small fiber neuropathy. Clin Neurol Neurosurg.

[11] Rolke R, Baron R, Maier C, Tolle TR, Treede R-D, Beyer A (2006). Quantitative sensory testing in the German Research Network on Neuropathic Pain (DFNS):standardized protocol and reference values. Pain.

[12] Forssell H, Jääskeläinen S, Tenovuo O, Hinkka S (2002). Sensory dysfunction in burning mouth syndrome. Pain.

[13] Kaplan I, Levin T, Alexandru D (2011). Thermal sensory and pain thresholds in the tongue and chin change with age, but are not altered in burning mouth syndrome. Skin Res Technol.

[14] Pigg M, Baad-Hansen L, Svensson P, Drangsholt M, List T (2010). Reliability of intraoral quantitative sensory testing (QST). Pain.

[15] Pigg M, Svensson P, List T (2011). Orofacial thermal thresholds:time-dependent variability and influence of spatial summation and test site. J Orofac Pain.

[16] Matos M, Wang K, Svensson P (2011). Quantitative Sensory Testing in the Trigeminal Region:Site and Gender Differences. J Orofac Pain.

[17] Baumgartner U, Magerl W, Klein T, Hopf HC, Treede RD (2002). Neurogenic hyperalgesia versus painful hypoalgesia: two distinct mechanisms of neuropathic pain. Pain.

[18] Zung WW, Satorius N, Ban TA (1986). Zung Self-Rating Depression Scale and Depression Status Inventory. Assessment of Depression.

[19] Lee HC, Chiu HF, Wing YK, Leung CM, Kwong PK, Chung DW (1994). The Zung Self-rating Depression Scale: screening for depression among the Hong Kong Chinese elderly. J Geriatr Psychiatry Neurol.

[20] Baad-Hansen L, Pigg M, Ivanovic SE, Faris H, List T, Drangsholt MT, Svensson P (2013). Chair-side intraoral qualitative somatosensory testing (QualST)—reliability and comparison between patients with atypical odontalgia and healthy control subjects. J Orofac Pain.

[21] Jääskeläinen S (2004). Clinical neurophysiology and quantitative sensory testing in the investigation of orofacial pain and sensory function. J Orofacial Pain.

[22] Puhakka AP, Forssell H, Soinila S, Laine MA, Jääskeläinen SK (2010). Burning mouth syndrome – a peripheral small fiber neuropathy. Clin Neurophysiol.

[23] Zakrzewska JM (2013). Multi-dimensionality of chronic pain of the oral cavity and face. J Headache Pain.

[24] Lauria G, Majorana A, Borgna M, Lombardi R, Penza P, Padovani A (2005). Trigeminal small-fiber sensory neuropathy causes burning mouth syndrome. Pain.

[25] Witt M, Reutter K (1998). Innervation of developing human taste buds. An immunohistochemical study. Histochem Cell Biol.

[26] Eppler CM, Hulmes JD, Wang JB, Johnson B, Corbett M, Luthin DR, Uhl GR, Linden J (1993). Purification and partial amino acid sequence of a mu opioid receptor from rat brain. J Biol Chem.

[27] Yilmaz Z, Renton T, Yiangou Y, Zakrzewska J, Chessell IP, Bountra C (2007). Burning mouth syndrome as a trigeminal small fibre neuropathy: increased heat and capsaicin receptor TRPV1 in nerve fibres correlates with pain score. J Clin Neurosci.

[28] Beneng K, Yilmaz Z, Yiangou Y, McParland H, Anand P, Renton T (2010). Sensory purinergic receptor P2X3 is elevated in burning mouth syndrome. Int J Oral Maxillofac Surg.

[29] Svensson P, Baad-Hansen L, Pigg M, List T, Eliav E, Ettlin D, Michelotti A, Tsukiyama Y, Matsuka Y, Jääskeläinen SK, Essick G, Greenspan JD, Drangsholt M (2011). Guidelines and recommendations for assessment of somatosensory function in oro-facial pain conditions--a taskforce report. J Oral Rehabil.

